# Biliary tract malignancies in HIV infected persons: a case series

**DOI:** 10.1186/1471-2334-14-S3-E37

**Published:** 2014-05-27

**Authors:** Vinay Kulkarni, Ritu Parchure, Prasad Bhoite, Harshal Gadhikar

**Affiliations:** 1 Prayas Health Group, Pune-411004, India; 2 Deenanath Mangeshkar Hospital, Pune-411004, India

## Background

Malignancies of biliary tract have been rarely reported in HIV disease.

## Case series

We report 3 cases of biliary tract malignancy associated with HIV infection from Prayas Amrita clinic, an NGO based HIV clinic in Pune, India. Two of the cases were diagnosed with gall bladder malignancies (GBM) and the third one had cholangio carcinoma (CC). All presented with advanced stage of malignancy with metastases and had poor prognosis. All were males with median age at presentation being 47 years (range - 46-48 years) They were diagnosed to have HIV for a median duration of 102 months (range: 69-118 months) . Median nadir CD4 count was 77cells/cmm (range: 71–163 cell/cmm). They were on first line ART for median 63 months (range: 41–72 months). A patient with GBM was Hepatitis B co infected. Prior AIDS related events of anemia, immune thrombocytopenic purpura and tuberculosis were present in the patient with CC and progressive multifocal leukoencephalopathy was in one of the patients with GBM. Being managed in resource limited setting; the absence of other risk factors is corroborative and lacks evidence.

## Conclusion

These 3 cases of biliary tract malignancies were seen recently in a relatively short span of time. All were men in late forties, infected with HIV for a long time and had no other known risk factors. This could be a pointer towards changing scenario of malignancies among HIV infected patients associated with prolonged survival.

